# Supplementary material for: N-Doped Honeycomb-like Ag@N-Ti3C2Tx Foam for Electromagnetic Interference Shielding
Source: Nanomaterials (Basel). 2022 Aug 27;12(17):2967. doi: 10.3390/nano12172967 (PMC9457588; doi:10.3390/nano12172967)
Supplement: Supplementary file 1 [file nanomaterials-12-02967-s001.zip › nanomaterials-1870418-supplementary.pdf]

Supporting Information

N-Doped Honeycomb-like Ag@N-$Ti_3C_2T_x$ Foam for Electromagnetic Interference Shielding

The following formulas were used for the calculation of the EMIS performance [1]:

$$R = |S_{11}|^2 = |S_{22}|^2 \quad (1)$$

$$T = |S_{12}|^2 = |S_{21}|^2 \quad (2)$$

$$R+A+T=1 \quad (3)$$

SE_R (Shielding by Reflection) = $10\log_{10}\left[\frac{1}{1-|S_{11}|^2}\right]$ (4)

SE_A (Shielding by Absorption) = $10\log_{10}\left[\frac{1-|S_{11}|^2}{|S_{12}|^2}\right]$ (5)

SE_T (Total Shielding effectiveness) = SE_R+ SE_A (6)

Transmissivity: $\frac{P_T}{P_I} = |S_{21}|^2$ (7)

Reflectivity: $\frac{P_R}{P_I} = |S_{11}|^2$ (8)

Absorptivity: $\frac{P_A}{P_I} = 1 - \frac{P_T}{P_I} - \frac{P_R}{P_I}$ (9)

where R represents the reflection ability of the material for electromagnetic waves and A represents the absorption ability of the material for electromagnetic waves.

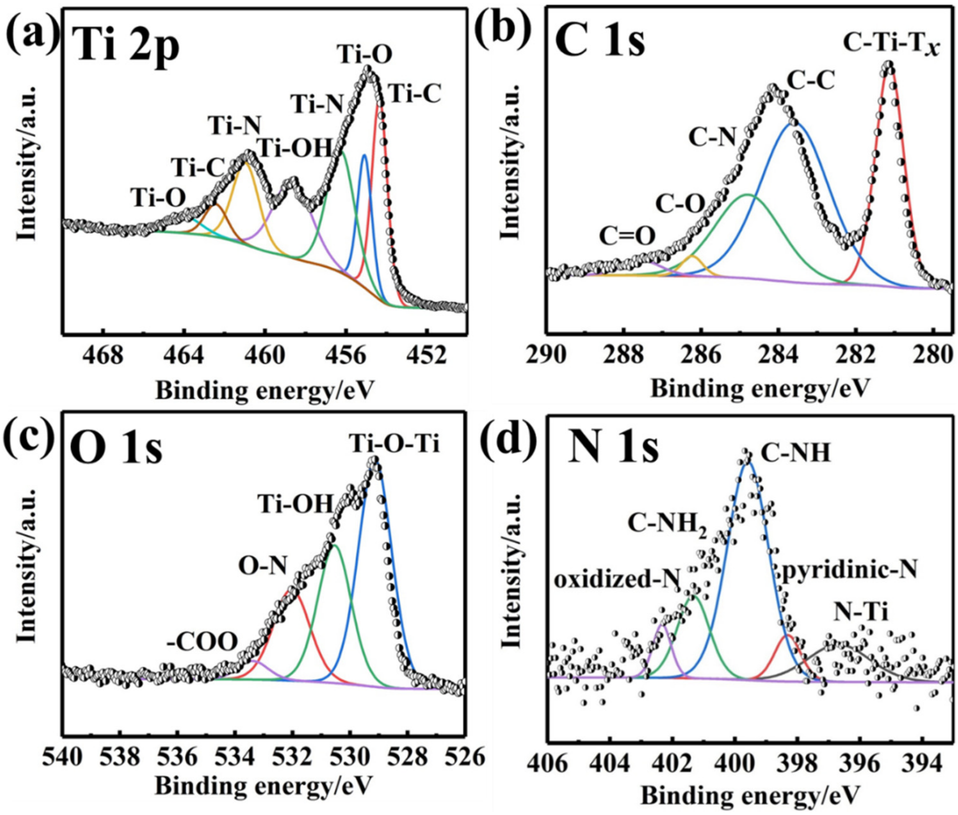

Figure S1. High-resolution XPS spectra of Ti 2p (**a**), C 1s (**b**), O 1s (**c**) and N 1s (**d**) of N-$Ti_3C_2T_x$ composites.

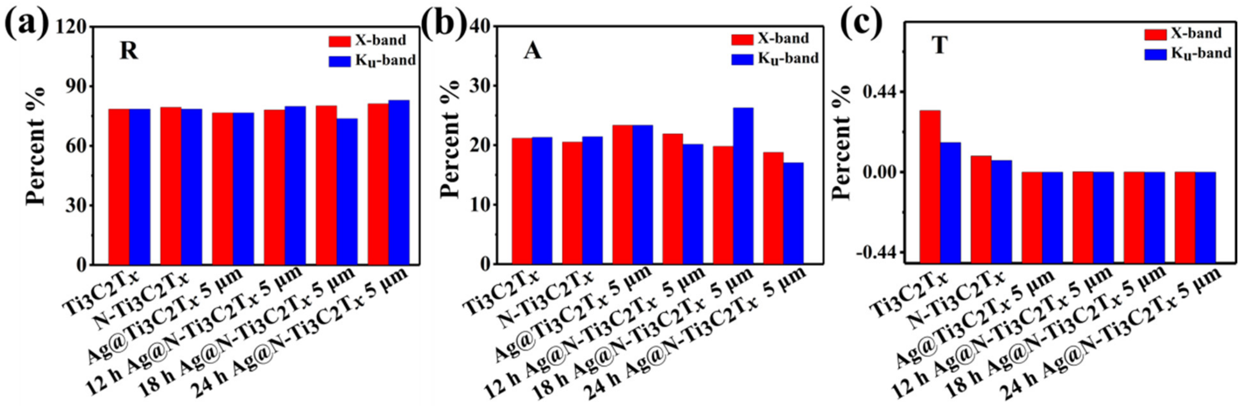

Figure S2. The (**a**) reflectivity, (**b**) absorptivity and (**c**) transmittivity of $Ti_3C_2T_x$, N-$Ti_3C_2T_x$ and Ag@N-$Ti_3C_2T_x$ composites.

Reference

[1] K, Raagulan.; R, Braveenth.; H,J, Jang.; Y, Seon Lee.; C,M, Yang.; B, Mi Kim.; J,J, Moon.; K,Y, Chai. Electromagnetic Shielding by MXene-Graphene-PVDF Composite with Hydrophobic, Lightweight and Flexible Graphene Coated Fabric. *Materials* (Basel) **2018**, *11*, 1803.
